# Adaptation to Aerobic Environment of *Lactobacillus johnsonii/gasseri* Strains

**DOI:** 10.3389/fmicb.2018.00157

**Published:** 2018-02-09

**Authors:** Diamante Maresca, Teresa Zotta, Gianluigi Mauriello

**Affiliations:** ^1^Department of Agricultural Sciences, University of Naples Federico II, Portici, Italy; ^2^Institute of Food Science, National Research Council, Avellino, Italy

**Keywords:** oxidative stress response, probiotic bacteria, antimicrobial activity, lactic acid bacteria, respiration metabolism

## Abstract

Oxygen is considered one of the main factors affecting probiotic bacteria survival due to the induction of oxidative damages caused by the action of reactive oxygen species (ROS). It has been shown that oxidative stress resistance in lactic acid bacteria is strongly dependent on the type of cell metabolism. Shift from fermentative to respiratory metabolism (through the addition of heme and menaquinone and in presence of oxygen) was associated to increase in biomass, long-term survival, and production of antioxidant enzymes. The aim of this work was to investigate the effect of aerobic (presence of oxygen) and respiratory (presence of oxygen, heme, and menaquinone) cultivation on the growth kinetic, catalase production, oxygen uptake, and oxidative stress response of *Lactobacillus johnsonii/gasseri* strains previously isolated from infant feces. Seven strains showed to consume oxygen under aerobic and respiratory conditions. The strain AL5 showed a catalase activity in both growth conditions, while AL3 showed this activity only in respiratory condition. Respiratory condition improved their tolerance to oxidative compounds (hydrogen peroxide and ROS generators) and further they showed promising probiotic features. The exploration of respiratory competent phenotypes with probiotic features may be extremely useful for the development of competitive starter or probiotic cultures.

## Introduction

The greatest challenge of the probiotic bacteria is enduring stresses encountered during food processing and gastrointestinal transit (Mills et al., 2011). Probiotic performances and robustness can be compromised by exposure to various environmental stresses, including acid, cold, drying, starvation, oxidative, and osmotic stresses, that may affect the physiological status and the functional properties of bacterial cells (Zhang et al., 2013). Survival to harsh conditions is an essential prerequisite for probiotic bacteria before reaching the target site where they can exert their health promoting effects (Expert Committee FAO/WHO, 2002). Several probiotics, in fact, have shown a poor resistance to technological processes, limiting their use to a restricted number of food products. The presence of oxygen is considered one of the main factors affecting the survival of probiotic lactic acid bacteria (LAB), anaerobic aerotolerant microorganisms, which lack the capability to synthesize an active electron transport chain. The aerobic environment in LAB may induce the production of toxic oxygen by-products (reactive oxygen species, ROS, such as superoxide anion radical, hydroxyl radical, and hydrogen peroxide) that may damage DNA, proteins, and lipids, resulting in cellular death (Amaretti et al., 2013). Moreover, hydrogen peroxide can further react with some cations (Fe^2+^ and Cu^2+^) leading to highly reactive oxidants via Fenton reaction (De Angelis and Gobetti, 2004; Kang et al., 2013). LAB get their energy mainly through substrate level phosphorylation, and lack both heme containing enzymes and active cytochrome oxidases, which are essential components for oxygen-linked energy metabolism (Kang et al., 2013). However, most LAB can grow under aerobic conditions and their simplest way to utilize oxygen is through the action of flavoprotein oxidases (NADH oxidase, NOX; pyruvate oxidase, POX; lactate oxidase, LOX; α-glycerophosphate oxidase, and L-amino acid oxidase) that use substrates such as pyruvate or NADH (Yamamoto et al., 2011). The activity of these enzymes, however, may result in the accumulation of toxic H_2_O_2_. LAB can prevent the oxidative damage by producing several ROS-degrading enzymes [catalase, superoxide dismutase (SOD), flavin-dependent oxidase, and peroxidases] and different redox and repair systems (glutathione and thioredoxin systems) and antioxidant enzymes [catalase, pseudo-catalase, SOD, and NADH peroxidases (NPR)] (Rochat et al., 2006; Kullisaar et al., 2010; Ruiz et al., 2011; Amaretti et al., 2013). Several authors have demonstrated that oxidative stress resistance in some LAB species is dependent on the type of metabolism and that the shift from fermentative toward respiratory metabolisms may increase their growth, long-term survival and stress tolerance (Gaudu et al., 2002; Rezaiki et al., 2004; Pedersen et al., 2012; Guidone et al., 2013; Ianniello et al., 2015). These effects are likely associated to the activation of the electron transport chain by growing cells in presence of oxygen, heme, and menaquinone (vitamin K2). Heme is an essential cofactor in heme-dependent catalase and cytochrome *bd* oxidase (CydAB) synthesis (Pedersen et al., 2008). Catalase can degrade H_2_O_2_ and protect the cell from oxidative damage, while CydAB can catalyze the rapid reduction of oxygen to water as well as to increase the ATP production. Instead, menaquinone may act as a central respiratory chain component delivering electrons from reducers (such as NADH dehydrogenase) to terminal oxidases (such as CydAB) (Brooijmans et al., 2009). However, while respiration metabolism and oxidative stress response have been extensively studied in *Lactococcus lactis* (Zotta et al., 2014; Ianniello et al., 2015) and *Lactobacillus casei* (Miyoshi et al., 2003; Zotta et al., 2014; Ianniello et al., 2015) and *Lactobacillus plantarum* groups (Watanabe et al., 2012; Zotta et al., 2012, 2013; Guidone et al., 2013) limited data are available for the strains of *Lactobacillus johnsonii* and *Lactobacillus gasseri*. These species are genetically related and belong to *Lactobacillus delbrueckii* group (Sun et al., 2014). *L. johnsonii* and *L. gasseri* are reported as the dominant bacteria within the lactobacilli population in human gut and in vaginal microbiota and are described as strict anaerobes with fermentative energy metabolism (Pridmore et al., 2003). Current data on oxygen tolerance and oxidative stress response in *L. johnsonii* are limited to the probiotic strain NCC 533 (Hertzberger et al., 2013, 2014). These authors have observed that the endogenous production of H_2_O_2_ is the main cause of oxidative stress in *L. johnsonii* NCC 533 during its aerobic growth, even though the presence of oxygen relieves its carbon dioxide (CO_2_) and acetate dependence, compared to anaerobic growth. On the contrary, no data on the capability to activate a minimal respiratory chain are available for *L. johnsonii* and *L. gasseri* species. The aim of this work was to evaluate the effect of aerobic (presence of oxygen) and respiratory (presence of oxygen, heme, and menaquinone) cultivation on the growth ability of *L. gasseri* and *L. johnsonii* strains. Tolerance of oxidative stress and other functional features [i.e., survival to simulated oral-gastrointestinal transit (OGIT) and antimicrobial activity] were also evaluated in order to select new promising probiotic strains.

## Materials and Methods

### Strains and Culture Conditions

A pool of 145 isolates potentially belonging to *Lactobacillus* genus were previously isolated from infant feces at the University of Naples and used in this study. *L. johnsonii* DSM 10533^T^, *L. johnsonii* DSM 20533, *L. gasseri* DSM 20243^T^, *L. gasseri* DSM 20077, and *Lactobacillus rhamnosus* ATCC 53103 (commercially known as GG strain) were used as reference strains. All lactobacilli were routinely propagated in Weissella Medium Broth (WMB; Zotta et al., 2012) pH 6.8 or in MRS Agar and incubated in anaerobiosis at 37°C for 24 h. Nine strains belonging to potential pathogenic and spoilage species (**Table 2**) were used in antimicrobial activity tests.

### Molecular Characterization of Isolates

Genomic DNA was extracted using the Insta-Gene matrix (Bio-Rad, Milan, Italy) according to the manufacturer’s protocol, with some modifications. Briefly, two to three colonies of each microorganism were suspended in 0.05 M phosphate-buffered saline (PBS) pH 7.0 and centrifuged for 1 min at 10,000 *g*. Pellet was dissolved in 200 μl of InstaGene matrix and incubated at 56°C for 30 min (Thermomixer Comfort, Eppendorf). After vortexing for 10 s, sample was treated for 8 min at 100°C. Mixture was centrifuged at 10,000 *g* for 3 min and the resulting supernatant, containing the bacterial DNA, was used for PCR. Quality and quantity of DNA was assessed using a NanoDrop spectrophotometer 1000 (Thermo Scientific, Milan, Italy).

In order to avoid the presence of clones, the isolates were firstly analyzed by rep-PCR using oligonucleotide GTG_5_ (5′-GTG GTG GTG GTG GTG-3′) primer (Invitrogen, Life Technologies, Milan, Italy). The reaction was performed in 20 μl mixtures containing: 50 ng DNA template, 2.5 μl of 10× PCR Buffer (Invitrogen, Milan, Italy), 50 mM MgCl_2_, 10 mM dNTPs mix, 10 μM primer, and *Taq* Polymerase (Bio-Rad) 5 U/μl. PCR was carried out using an initial denaturation step at 95°C for 4 min, followed by 35 cycles of 1 min at 94°C, 1 min at 40°C, and 1 min at 72°C each, and by a final extension of 8 min at 72°C. PCR products were separated by electrophoresis (3 h at 130 V) on 1.7% (w/v) agarose gel stained with 0.1 μl/ml SYBR safe (Invitrogen) and visualized by UV transillumination. Rep-PCR profiles were analyzed by BioNumerics 5.0 software (Applied Maths) using Pearson’s correlation coefficient with UPGMA (Unweighted Pair Group Method with Arithmetic Mean) clustering of averaged profile similarities.

Universal primers (Invitrogen) fD1 (5′-AGAGTTTGATCCTGGCTCAG-3′) and rD1 (5′-AAGGAGGTGATCCAGCC-3′) were used to amplify the 16S rRNA gene of isolates. PCR reaction mixture (final volume 50 μl) contained 50 ng of DNA template, 5 μl of 10× buffer (200 mM Tris–HCl pH 8.4, 500 mM KCl), 25 mM MgCl_2_, 10 mM dNTPs mix, primers 50 pM, and *Taq* Polymerase 5 U/μl. PCR amplification was performed using an initial denaturation step at 95°C for 3 min, followed by 30 cycles of 45 s at 94°C, 45 s at 55°C and 1 min at 72°C each, and by a final extension of 5 min at 72°C. The PCR products were separated by on agarose gel 1.5% (w/v), containing 0.1 μl/l SYBR safe, purified using QIAquick PCR Purification Kit (Qiagen, Milan, Italy), and sequenced by Primm srl (Milan, Italy). Research for DNA similarity was performed using the BLAST program of the National Center for Biotechnology Information (NCBI^1^) GenBank. Strains showing a % similarity higher than 98% with *L. johnsonii/gasseri* were used for further analyses.

### Preliminary Evaluation of Probiotic Potential of Isolates

The ability of *L. johnsonii/gasseri* strains to survive in simulated OGIT was performed according to Vizoso et al. (2006) with some modifications. Briefly, overnight cultures were recovered by centrifugation (6500 *g* for 10 min), washed twice with sterile saline (NaCl 0.85%) and suspended in equal volume of simulated saliva juice (SSJ: NaCl 5 g/l, KCl 2.2 g/l, CaCl_2_ 0.22 g/l, NaHCO_3_ 1.2 g/l, and lysozyme 100 mg/l, pH 6.9) and incubated for 5 min at 37°C. The suspension was then centrifuged as above, re-suspended in an equal volume of simulated gastric juice (SGJ: NaCl 5 g/l, KCl 2.2 g/l, CaCl_2_ 0.22 g/l, NaHCO_3_ 1.2 g/l, and pepsin 3 g/l, pH 2.5) and incubated at 37°C for 120 min under gentle agitation (200 rpm) to simulate peristalsis. After centrifugation, pellet was re-suspended in an equal volume of simulated pancreatic juice (SPJ: NaHCO_3_ 6.4 g/l, KCl 0.239 g/l, NaCl 1.28 g/l, 0.5% bile salts, and 0.1% pancreatin, pH 7.0) and incubated at the same condition of SGJ. Survival (%) was calculated after each treatment. *L. rhamnosus* GG was used as positive control in this experiment.

Antimicrobial activity was assayed using an agar spot test and a well diffusion agar test, as previously described by Banerjee et al. (2013). In the first case, 10 μl of each overnight culture were spotted onto a MRS Agar plate. After incubation at 37°C for 24 h, plate was overlaid with 10 ml of Tryptone Soya Broth (TSB, Oxoid, Milan, Italy) supplemented with 0.75% agar (TSB soft agar) previously inoculated with the indicator strains (**Table 2**) to reach a final concentration of 1 × 10^6^ CFU/ml. After 24 h of incubation at optimal growth temperature of indicator strains the antimicrobial activity was detected by the presence of a clear growth inhibition zone around the colony of tested strain. In the well diffusion agar test, a cell free supernatant was recovered by centrifugation (6500 *g* for 10 min), adjusted at pH 6.5 with NaOH 1 M, heat-treated for 10 min at 80°C and sterilized by a low-binding protein 0.22 μm pore size filter. Fifty microliters of supernatant were placed into a 6 mm-diameter well, done in a TSB soft agar plate previously inoculated with the indicator strain at a final concentration of 1 × 10^6^ CFU/ml. After incubation at optimal growth temperature of indicator strain, the antimicrobial activity was determined by measuring the diameter (cm) of the inhibition zone around the wells. In both agar spot test and a well diffusion agar test results were calculated as the mean of three experiments. The strains that showed antimicrobial activity in well diffusion agar test were further tested to investigate the nature of the antimicrobial substance produced. The sensitivity to different enzymes was tested using 10 mg/ml (final solution) of lipase, catalase, papain, trypsin, α-chymotrypsin, pronase E, and pepsin in PBS. Ten microliters of solution used in well diffusion assay were spotted onto TSB soft agar plates previously inoculated with the indicator strain. Afterward, 8 μl of enzyme solution were deposited adjacent the spot of supernatant to inhibit the activity of antimicrobial substance.

### Aerobic and Respiratory Growth and Catalase Production

The assessment of aerobic and respiratory growth as well as the presence of catalase activity were performed as reported by Zotta et al. (2014). Strains were cultivated for 24 h at 37°C in 24-well microplates in different growth conditions: anaerobic (AN, static cultivation in modified WMB with 10 g/l of glucose, pH 6.8, with AnaeroGen bags, Oxoid), aerobic (AE, in modified WMB, shaken on a rotary shaker at 150 rpm), and respiratory (RS, AE growth in modified WMB, supplemented with 2.5 μg/ml of hemin and 1 μg/ml of menaquinone) cultivations. Micro-plates were inoculated (2% v/v) with standardized [optical density at 650 nm (OD_650_) = 1.0] overnight anaerobic pre-cultures and incubated for 24 h at 37°C. At the end of incubation, the OD and pH values were measured. Catalase activity was qualitatively evaluated by re-suspending the washed biomass derived from 1 ml of culture in all different conditions (AN, AE, and RS) in 100 μl of a 3% (v/v) H_2_O_2_ solution. Catalase production was revealed by an evident formation of bubbles in the cell suspension. Three independent replicates were carried out for each experiment.

Strains that showed H_2_O_2_-degrading capability were further tested to quantify the enzymatic activity. Five milliliters of AN, AE, and RS cultures standardized at OD_650_ = 1 were centrifuged at 13,000 *g* for 5 min and the resulting pellet mixed with 1.0 ml of 60 mM H_2_O_2_ in 50 mM PBS pH 7. The activity was measured at 240 nm after 3 min incubation at room temperature. Results were expressed as units (U) per ml of solution and calculated according to the following formula:

U=(ΔOD×ε×df)/(t×v)

where,

ΔOD: decrease in absorbance after 3 min at 240 nm; 𝜀: 39.4 M^-1^/cm^-1^, extinction coefficient of H_2_O_2_ at 240 nm; *df*: dilution factor; *t*: time of analysis in min; *v*: volume of sample.

### Oxygen Uptake

The consumption of oxygen in AN, AE, and RS growing cells was measured as described by Ricciardi et al. (2014). Briefly, washed and standardized (OD_650_ = 1) biomass was re-suspended in air-saturated solution of 5.5 mM glucose and 0.002 g/l of resazurin sodium salt (Sigma) in 0.1 M PBS pH 7. The discoloration time (DT, expressed in minutes) from blue oxidized form (resazurin) to colorless reduced form (dihydroresorufin) was used as indicator of oxygen uptake. The strain *Pseudomonas fragi* SP1 was used as positive control.

### Effect of Aerobic and Respiratory Cultivation on Oxidative Stress Tolerance

AN, AE, and RS cultures, washed twice and standardized (OD_650_ = 1) in 0.1 M PBS pH 7.0, were loaded in 96-well microplates and exposed to different concentrations (from 0.62 to 320 mM serial twofold dilution in 0.1 M PBS pH 7.0) of H_2_O_2_, menadione and pyrogallol for 30 min at 37°C in anaerobiosis. Stressed cultures were inoculated (10% v/v) in sterile WMB broth (20 g/l glucose, pH 6.8; 96-well microplates) and the surviving cells were detected by evaluating the medium turbidity (presence/absence) after 24 h of incubation at 37°C in anaerobic conditions. For each strain the results were expressed as maximum tolerated concentration (mM) of oxidative stress compounds (**Table 4**).

### *In Silico* Analysis of Genes Involved in Aerobic-Respiratory Pathway and Oxidative Stress Response

The presence of genes coding for the main enzymes involved in oxygen utilization (pyruvate oxidase, *pox*; acetate kinase, *ack*; lactate oxidase, *lox*; NADH oxidase, *nox*), electron transport chain (NADH dehydrogenase, *ndh*; cytochrome *bd* oxidase, *cydABCD* operon; menaquinone biosynthesis complex, *menFDXBEC*, and ubiquinone/menaquinone biosynthesis methyltransferase, *ubiE*) and oxidative stress response (heme- and manganese-dependent catalases, *kat* and *Mnkat*; manganese-dependent SOD, *sodA*; thioredoxin/thioredoxin reductase system, *trx-trxB*; glutathione peroxidase/reductase system, *gop* and *gor*; NADH peroxidase, *npr*) was evaluated in the finished, draft and permanent draft genomes of *L. johnsonii* and *L. gasseri* from IMG/M database^2^ (Supplementary Table S1). The occurrence expressed in % (Occ) of each gene in the *L. johnsonii* and *L. gasseri* group was calculated. The sequences retrieved from the genomes of *L. lactis* subsp. *cremoris* MG1363 (*menFDXBEC*), *L. plantarum* WCFS1 (*pox*, *ack*, *lox*, *nox*, *npr*, *cydABCD*, *ubiE*, *kat*, *trxA-trxB*, *gop-gor*), *L. plantarum* ATCC 14431 (*Mn-kat*), and *Lactobacillus sakei* 23K (*sodA*) were used as queries. Sequence similarity was detected using the default cut-off parameter (% of identity) obtained by ClustalW2 multiple sequence alignment tool.

## Results

### Molecular Characterization of Strains Belonging to *L. johnsonii/gasseri* Species

On the basis of genotypic (rep-PCR analysis) and phenotypic (i.e., cell and colony morphology, growth performance, planktonic or aggregated growth in MRS broth) characteristics, a total of 80 strains were selected and subjected to 16S rRNA gene sequencing analysis. Results of BLAST analysis showed that 55 strains belonging to *L. johnsonii/gasseri* species (≥98% homology), while the remaining 25 strains to *L. casei* (15 strains) and *L. plantarum* groups (10 strains). Since 16S rRNA gene sequence is not discriminative for *L. johnsonii* and *L. gasseri* species, in all experiments we decided to indicate the strains as *L. johnsonii*/*gasseri*. On the basis of the different rep-PCR profiles, 34 strains of *L. johnsonii/gasseri* were selected and used to investigate the potential probiotic features and the capability to grow in presence of oxygen and oxidative stress conditions.

### Survival to Simulated OGIT

Results of viable counts of the strains after simulated OGIT showed that all strains were significantly (*P* < 0.05) resistant (at least the 50% of initial population) to SSJ exposure (**Table 1**). On the contrary, the number of survivors to SGJ and SPJ dramatically decreased. Only 13 and 6 strains had at least 50% of viable cells (*P* < 0.05) after the exposure to SGJ and SPJ, respectively (**Table 1**). Specifically, the six strains tolerant of SPJ showed more than 90% of survival. As expected, the probiotic strain *L. rhamnosus* GG showed a high resistance to OGIT (>90% of final survival).

**Table 1:** Strains showing a survival ≥50% after exposure to the different conditions of oral-gastrointestinal transit.

SSJ	SGJ	SPJ
All strains	AL5, BR32, ID5AN, ID7AN, AL8, ALJ, AL3, BM1CM, BM1CP, BR35, AL15, *L. johnsonii* 10533^T^, *L. gasseri* 20243^T^	AL5, BR32, ID5AN, ID7AN, AL3, *L. johnsonii* 10533^T^

### Antimicrobial Activity

Results of agar spot test showed that 22 strains of *L. johnsonii*/*gasseri* exhibited antimicrobial activity against at least two of the nine indicator strains used in this study (data not shown). In **Table 2** are reported the strains with the widest inhibitory pattern. The strains AL5, AL3, and ALA had the highest antimicrobial activity against the most of indicators (diameter of inhibition halos >2 cm). On the other hand, results of well-diffusion agar assay showed that only the strains BM4, AL5, BR32, AL3, ID5AN, BM1CM, and BM61CG produced antimicrobial substances, although they inhibited only *Staphylococcus aureus* DSM 20231 (data not shown). Inhibition was not associated to the production of bacteriocin-like substances or H_2_O_2_ since the antagonistic activities were not affect by the action of proteolytic enzymes and catalase.

**Table 2:** Results of agar spot test on the strains showing a remarkable antimicrobial activity against the selected indicator strains.

Indicators									

Strains	*Brochothrix thermosphacta* ATCC 11509	*Brochothrix thermosphacta* 7R1^a^	*Pseudomonas fragi* 6P2^a^	*Listeria monocytogenes* ATCC 7644	*Listeria innocua* ATCC 1770	*Micrococcus luteus* ATCC 10240	*Bacillus subtilis* DSM 5547	*Staphylococcus aureus* DSM 20231	*Escherichia coli* 1634^a^
BM4	+	++	-	++	++	++	+	++	+
AL5	+++	+++	++	+++	+++	+++	+	++	-
AL9	+	+	+	++	+++	+	+	-	+
BR32	++	++	+++	++	+	++	+	+	-
AL3	++	+++	+++	+++	+++	++	+	+++	+
ALA	+++	+++	+++	+++	+++	+++	+++	+	-
ID5AN	+	+	+	++	+	+	+	+	-
10533^T^	+	+	-	++	++	-	+	+	+

*^a^Provided by Division of Microbiology, Department of Agriculture, University of Naples Federico II. -, no inhibition halo; +, inhibition haloes ≥0.5 and ≤1.5 cm; ++, inhibition haloes >1.5 and ≤2.0 cm; +++, inhibition haloes >2.0 cm.*

### Aerobic and Respiratory Promoting Growth, Oxygen Uptake, and Catalase Production

**FIGURE 1:**
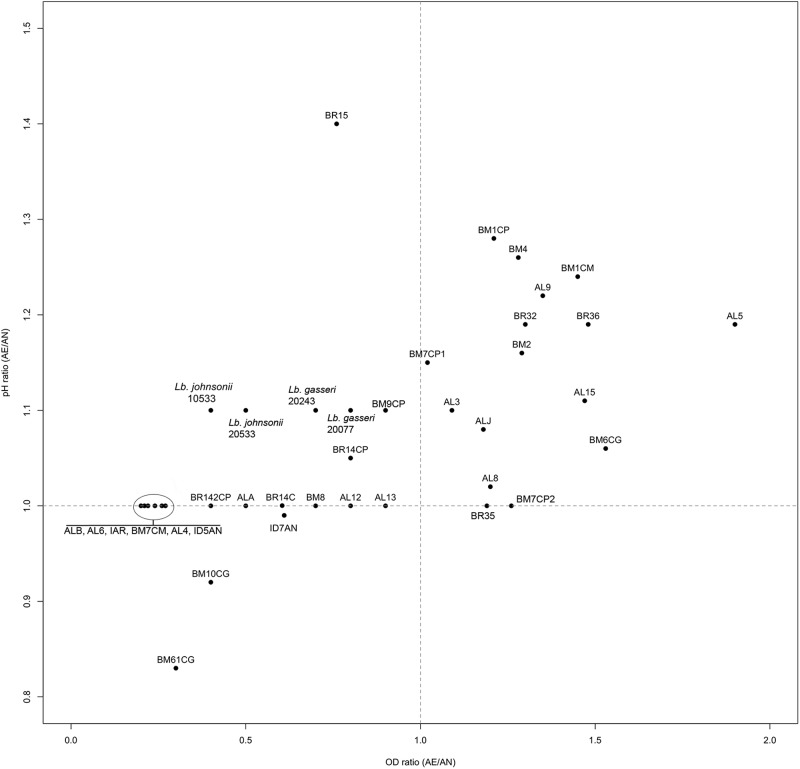
Scatter plot of OD_650_ ratio against pH ratio in AE/AN of *L. johnsonii/gasseri* strains.

**FIGURE 2:**
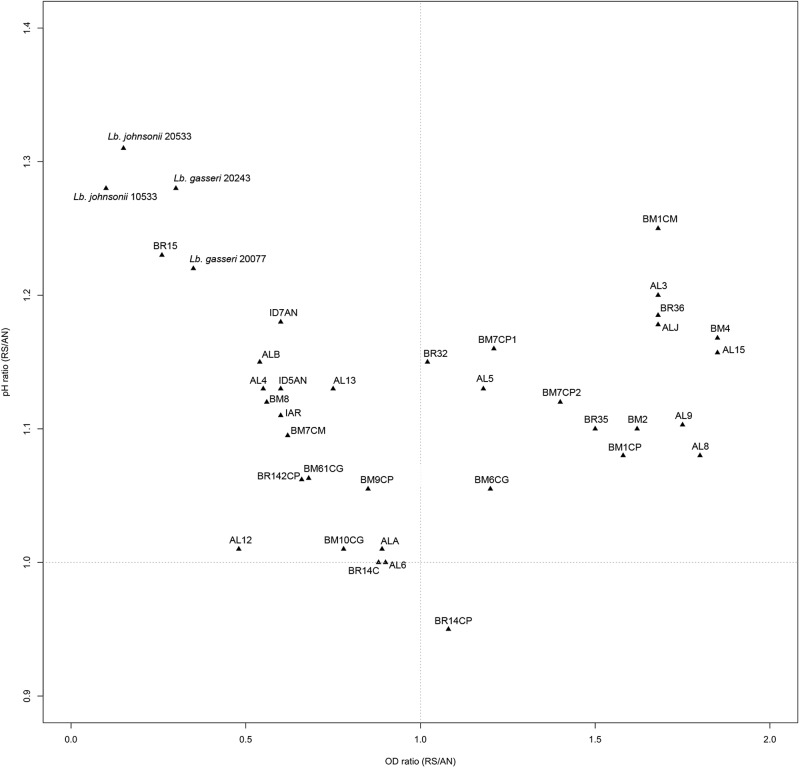
Scatter plot of OD_650_ ratio against pH ratio in RS/AN of *L. johnsonii/gasseri* strains.

**FIGURE 3:**
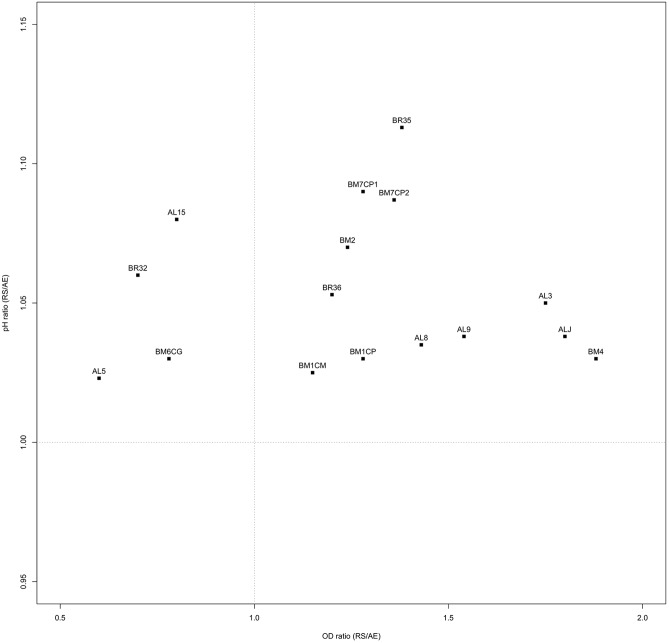
Scatter plot of OD_650_ ratio against pH ratio in RS/AE of *L. johnsonii/gasseri* strains.

Ratios between OD and pH values (AE/AN, RS/AN) measured in the different growth conditions were calculated and used to identify the phenotype of each strain. Specifically, when both OD and pH ratios in AE/AN were >1 (**Figure 1**, upper right side), strains were indicated as oxygen tolerant phenotypes (OTP); when OD and pH ratios in RS/AN were >1 (**Figure 2**, upper right side), strains were indicated as respiration-competent phenotypes (RCP). Results showed that 14 strains had both OTP and RCP, while two strains had only the RCP. For these 16 strains, the OD and pH ratios in RS/AE were also calculated (**Figure 3**) and the oxygen uptake was tested. Results showed that 12 strains grew better when hemin and menaquinone were supplied (**Figure 3**, upper right side), while four strains grew better when only oxygen was supplied (**Figure 3**, upper left side). Interestingly, the four strains showing OD ratio RS/AE <1 (AL5, AL15, BM32, and BM6CG) and the three strains showing the best growth performance in RS (ALJ, AL3, and BM4) resulted to be the strains that consumed oxygen during their growth (DT < 180 min, **Table 3**). Consistently, ALJ, AL3, and BM4 consumed more oxygen in RS than in AE, AL5 consumed more oxygen in AE and BM6CG, unexpectedly, consumed oxygen only in AE cultivation. Instead, AL15 and BM32 showed the same DT in both AE and RS (**Table 3**). Catalase activity was detected only in two strains. Specifically, AL5 showed 11.30, 12.20, and 12.00 U of enzymatic activity in AN, AE, and RS, respectively, while AL3 showed catalase activity only in RS with 13.50 U.

**Table 3:** Consumption of oxygen in *Lactobacillus johnsonii*/*gasseri* strains expressed as discoloration time in minutes (DT) of the redox indicator resazurin.

Strains	Growth conditions		
	AN	AE	RS
AL9	>180	>180	>180
AL5	>180	90	120
AL15	>180	100	100
AL8	>180	>180	>180
BR32	>180	100	100
BM4	>180	130	75
BM7CP2	>180	>180	>180
BR35	>180	>180	>180
BM6CG	>180	120	>180
BM1CP	>180	>180	>180
BR36	>180	>180	>180
BM2	>180	>180	>180
BM7CP1	>180	>180	>180
BM1CM	>180	>180	>180
ALJ	>180	120	100
AL3	>180	110	75
*Pseudomonas fragi* SP1	ND	65	ND

*AN, anaerobiosis; AE, aerobiosis; RS, AE growth supplemented with 2.5 μg/ml of hemin and 1 μg/ml of menaquinone.*

### Effect of Aerobic and Respiratory Conditions on the Oxidative Stress Tolerance

In order to investigate the effect of growth conditions on oxidative stress tolerance the strains cultivated in AN, AE, and RS conditions were exposed to generators of superoxide anion (menadione and pyrogallol) and H_2_O_2_. Strains cultivated in AE exhibited the lowest tolerance of all oxidative compounds (**Table 4**). For several strains, the respiratory growth increased the resistance to H_2_O_2._ Most of respiratory growing cultures had H_2_O_2_-tolerance similar to that of cells grown under anaerobic conditions, while only AL9 had a lower resistance when cultivated in presence of oxygen, hemin, and menaquinone. Compared to anaerobic conditions, respiration promoted the menadione resistance only in five strains and impaired the survival in AL9, BM6CG, and BM1CM. With exception of AL9, the resistance of respiratory cultures to pyrogallol was similar to that of anaerobically growing cells.

**Table 4:** Tolerance of H_2_O_2_, menadione and pyrogallol (expressed as maximum tolerated concentration; mM) in *L. johnsonii/gasseri* strains grown under AN, AE, and RS conditions.

Strains	H_2_O_2_ (mM)			Menadione (mM)			Pyrogallol (mM)		
	AN	AE	RS	AN	AE	RS	AN	AE	RS
AL9	20	0	1.25	10	0	0	160	10	10
AL5	10	10	40	2.5	5	20	320	80	320
AL15	20	10	40	0.62	1.25	5	320	80	320
AL8	20	5	20	5	0	5	80	2.5	80
BR32	20	2.5	20	1.25	1.25	5	80	40	80
BM4	20	20	20	5	2.5	5	320	80	320
BM7CP2	20	5	20	2.5	0.62	2.5	80	20	80
BR35	20	5	20	5	0	2.5	80	20	80
BM6CG	20	0	20	10	0	5	80	0	80
BM1CP	20	5	20	5	1.25	5	80	2.5	80
BR36	10	5	40	10	5	20	80	20	80
BM2	20	10	40	2.5	2.5	2.5	320	40	80
BM7CP1	20	10	20	2.5	2.5	2.5	80	40	80
BM1CM	20	2.5	20	10	0	5	80	0	80
ALJ	5	2.5	20	2.5	0	2.5	40	40	80
AL3	10	10	40	2.5	0	20	80	40	80

*Growth conditions: AN, anaerobiosis; AE, aerobiosis; RS, AE growth supplemented with 2.5 μg/ml of hemin and 1 μg/ml of menaquinone.*

### *In Silico* Analysis of Genes Involved in Aerobic-Respiratory Metabolism and Oxidative Stress Response (Supplementary Table S1)

Results of *in silico* analysis showing the occurrence % of identity (% ID) of each gene are indicated in Supplementary Table S1. Interestingly, we found 100% of occurrence of genes *pox*, *ack*, *cydA*, *cydB*, *ubE*, *trxA*, and *trxB* both in *L. johnsonii* and in *L. gasseri*. Instead, remaining genes were found at very low % in both species, with exclusion of *gor* gene that was found in all *L. johnsonii* genomes and lacking in only two *L. gasseri* genomes. Moreover, an ID in the range 45–55% was registered for most of genes. Finally, the genes *kat*, *Man-kat*, *sodA*, *menFDXBEC*, and *nox*, not reported in the table, have been never annotated in genomes of *L. johnsonii* and *L. gasseri*.

## Discussion

This study investigated the adaptive response of promising probiotic *L. johnsonii/gasseri* strains to switch from fermentative to aerobic and respiratory metabolism and the effect of this metabolic pathway on oxidative stress response. We found that about 70% of lactobacilli isolated from baby stools belonged to *L. johnsonii/gasseri* species. This result is in agreement with findings of many authors (Wall et al., 2006; Mitsou et al., 2008; Morelli et al., 2008), who demonstrated that *L. johnsonii/gasseri* species are the more commonly homofermentative lactobacilli isolated in newborns and infant feces. According to the FAO/WHO definition, probiotics are “live microorganisms which, when administered in adequate amounts, confer a health benefit on the host” (Expert Committee FAO/WHO, 2002). In order to perform their physiological role, probiotics bacteria must overcome a number of stresses before they reach the target site (Kwarteng et al., 2015). The ability to survive the gastrointestinal transit and the antimicrobial activity are two important features of probiotics (Vizoso et al., 2006). In this study, 34 *L. johnsonii/gasseri* strains were screened for the ability to pass through OGIT. All strains showed a strong resistance to saliva juice, suggesting a high ability to survive in the presence of lysozyme. On the contrary, for most strains the viability decreased when exposed to simulated gastric and intestinal juice. Six strains, however, showed a great resistance to OGIT, with levels of survival comparable to those of *L. rhamnosus* GG, suggesting their possible use as probiotic supplements.

The capability to inhibit the growth of pathogenic and spoilage bacteria varied among *L. johnsonii/gasseri* strains. The inhibitory activity demonstrated with agar spot test could be due to a lowering of pH due to organic acids production; indeed, it disappeared when free cell supernatants were neutralized. However, the ability to produce organic acids should be a useful feature to reduce colonization of pathogenic microorganisms in human GIT. Tejero-Sariñena et al. (2012) showed that the production of organic acids by different probiotic strains reduced the growth of potential pathogenic microorganisms. In presence of low O_2_ concentration, acetic acid and H_2_O_2_ may be produced by some probiotic strains. Pridmore et al. (2008) described the ability of some *L. johnsonii* and *L. gasseri* strains to produce H_2_O_2_ and acetic acid, with antimicrobial activity against *Salmonella* Typhimurium SLI344. In this study, the neutralized cell-free supernatants had inhibitory activity against *S. aureus*. Since bacteriocin-like and H_2_O_2_ activities were excluded, we hypothesized that the inhibition may be due to the production of neutral compounds or some undissociated short chain free fatty acids with antimicrobial activity (Huang et al., 2011; Ricke, 2014; Aldunate et al., 2015). However, further investigations are needed to reveal the nature of antimicrobial substances produced by *L. johnsonii* and *L. gasseri* active against *S. aureus*.

A large diversity on the capability to grow in presence of oxygen and/or respiratory cofactors was present within the *L. johnsonii* and *L. gasseri* strains and on the basis of growth performances and oxygen consumption it was possible to group the strains in: (i) oxygen-tolerant anaerobes, capable to grow in aerobic and respiratory conditions, but not able to consume oxygen (i.e., AL8, AL9, BM2, BM1CM, BM1CP, BM7CP1, BM7CP2, BR35, and BR36); (ii) aerobic phenotypes, able to consume oxygen and for which aerobiosis was the best growth condition compared to respiration (i.e., AL5, AL15, BM32, and BM6CG); (iii) respiratory phenotypes, able to consume oxygen and for which respiration was the best growth condition compared to aerobiosis (i.e., ALJ, AL3, and BM4); (iv) oxygen-sensitive anaerobes, unable to grow in both aerobic and respiratory conditions (remaining strains). Hertzberger et al. (2013) evaluated for the first time the response of *L. johnsonii* NCC 533 to oxidative conditions. Compared to anaerobiosis, the presence of oxygen relieved the acetate and CO_2_ growth dependencies of the strain, but induced more rapidly the entry in stationary phase. In this study, we found four strains (AL5, BM6CG, AL15, and BR32) with aerobic phenotype that showed increased biomass production, reduced acidification, and oxygen uptake capability, suggesting a possible activation of aerobic metabolism. In several LAB strains, the stimulatory effect of oxygen was shown to be dependent by POX and acetate kinase (ACK) activities (Goffin et al., 2006; Quatravaux et al., 2006). According to results of *in silico* analysis, *L. johnsonii* and *L. gasseri* possess genes predicted to encode for both POX and ACK (Occ = 100%), the main enzymes involved in aerobic pathway (Pridmore et al., 2008). In presence of oxygen and at low glucose concentration, pyruvate can be metabolized by POX–ACK pathway, allowing the production of acetate, CO_2_ and extra ATP biosynthesis (Pedersen et al., 2012). Therefore, in our strains the possible POX–ACK pathway activation could directly explain the observed physiological consequence, such as increase of pH due to a possible conversion of pyruvate into acetate and increase of OD due to extra ATP generation. As a matter of fact, Hertzberger et al. (2013) demonstrated that *pox* deletion in *L. johnsonii* NCC 533 resulted in a slower growth rate and a growth arrest upon CO_2_ depletion, confirming the positive role of POX–ACK pathway. The respiratory growth was never investigated in *L. johnsonii*/*gasseri* strains. Like the aerobic metabolism adaptation, the ability to shift toward respiratory pathway was strain-specific. In this study, we found that the strains AL3, ALJ, and BM4 had a respiratory phenotype. Like most LAB, the genomes of *L. johnsonii* and *L. gasseri* lack the genes for menaquinone and heme biosynthesis, but harbor those for the cytochrome oxidase production (Occ = 100%). Thus, in these species, the respiratory metabolism may occur only when heme and menaquinone are supplied (Pedersen et al., 2012). As well as in all lactobacilli, the mechanism of heme uptake is unknown. However, heme-binding proteins and heme homeostasis systems have been investigated in several LAB strains competent for aerobic respiration (Gaudu et al., 2003; Lechardeur et al., 2010, 2011; Sawai et al., 2012). In several LAB strains, the growth in presence of oxygen may result in the production and accumulation of H_2_O_2_ and ROS. Pridmore et al. (2008) and Hertzberger et al. (2013) demonstrated that the accumulation of H_2_O_2_ due to LOX and POX activity was the primary reason of oxidative stress in *L. johnsonii* NCC 533. Therefore, the ability to scavenge H_2_O_2_ and ROS may contribute to the survival in aerobic conditions. Surprisingly, we found that two strains showed a catalase like activity. Specifically, the strain AL5 in all growth conditions, while the strain AL3 only in RS conditions. Heme and manganese-catalase activity has been previously studied in several LAB species (Knauf et al., 1992; Rochat et al., 2006; Guidone et al., 2013; Ianniello et al., 2015, 2016) but this is the first study reporting catalase positive phenotype in *L. johnsonii/gasseri* strains. However, genes encoding for heme-catalase or Mn-catalase were never annotated in *L. johnsonii* and *L. gasseri* genomes. Heme-dependent and Mn-dependent catalase activities were previously found in the respiration-competent strain *L. casei* N87 (Zotta et al., 2014; Ianniello et al., 2015, 2016) and the genome sequence confirmed the presence of both genes (Zotta et al., 2016). We sequenced whole genome of the strains AL3 and AL5 (Maresca et al., 2017) and a first sequence analysis showed that both strains do not possess genes for heme- and manganese-catalase. However, these strains as well as the stains AL15, BR36, BM2, and ALJ showed a higher resistance to H_2_O_2_ and ROS generators when cultivated in RS, compared to AN and AE conditions. Results of genome analysis (Maresca et al., 2017) revealed that AL3 and AL5 genomes have sequences encoding for NOX and NPR, the main enzymes involved in the NAD(P)-dependent H_2_O_2_ scavenging pathway in LAB. On the other hand, the increased resistance to H_2_O_2_ under RS condition due to NPR activity was previously demonstrated in *L. rhamnosus* GG, *L. casei* Shirota and in some respiration-competent strains of *L. casei* (Ianniello et al., 2015). Moreover, all strains were able to cope to menadione and pyrogallol stress in both RS and AN condition. The genome analysis of AL3 and AL5 strains revealed the presence of large pattern of genes involved in oxidative stress resistance mechanisms (Maresca et al., 2017). Surprisingly, the results of genome analysis revealed that AL3 and AL5 genomes have sequences encoding for the antioxidant enzyme SOD, Dps-like peroxide resistance protein (DPR) and for the complete thioredoxin reductase system. Noteworthy, SOD sequences are relatively rare in *Lactobacillus* genus and occur only in some strains of *Lactobacillus sanfranciscensis*, *L. sakei*, *Lactobacillus curvatus*, and *Lactobacillus paracasei* species (Yamamoto et al., 2011; Zotta et al., 2017). This is the first time that *sod* gene was annotated in *L. gasseri* genome. Dpr is a member of the DNA-binding proteins from starved cells (Dps) that are able to provide cell protection during exposure to harsh environmental stress, including oxidative stress and nutritional deprivation. The function of Dpr has been the object of numerous studies and its role in acid and oxidative stress (iron and hydrogen peroxide detoxification) resistance in *Escherichia coli*, has been proposed (Calhoun and Kwon, 2011). While, regarding to LAB, the role of Dpr in the oxidative stress response was reported only in some species of *Streptococcus* and in *L. lactis* (Pulliainen et al., 2003; Cesselin et al., 2011). Finally, thioredoxin reductase system belong to flavoprotein disulfide oxidoreductases family and play a significant role in oxidative stress resistance, maintaining a high intracellular thiol/disulfide homeostasis in both prokaryotic and eukaryotic cells (Harel and Storz, 2000; Jänsch et al., 2007). Its role in oxygen and H_2_O_2_ tolerance have been explored in some *Lactobacillus* species (Li et al., 2003; Vido et al., 2005; Serrano et al., 2007; Jänsch et al., 2011; Serata et al., 2012). In conclusion, we investigated 34 *L. johnsonii*/*gasseri* strains from baby stools and found that some of them showed both typical probiotic features, like resistance to OGIT and antimicrobial activity, and aerobic environment adaptation. In particular, the strains AL5, BR32, and AL3 showed to tolerate very well the stress due to OGIT, in fact more than 90% of their population survived this treatment. Furthermore, they showed to inhibit the growth of most of indicator strains used in this study by producing organic acids, and the growth of a *S. aureus* strain by producing a sort of antimicrobial substance, not ascribable to a protein or to hydrogen peroxide, that will be further investigated. Additionally, they showed an interesting adaptation to aerobic environment with the AL5 and BR32 strains showing an OTP and the AL3 strain showing a RCP. Their overall enhanced resistance to oxidative stressors and the evidence of catalase production ability of AL5 and AL3 strains confirmed this adaptation. Probiotic strains of this type could effectively work both during biomass production and in food or processing in which the aerobic condition could be a limiting factor for a standard probiotic strain.

## Author Contributions

All authors listed have made a substantial, direct and intellectual contribution to the work, and approved it for publication.

## Conflict of Interest Statement

The authors declare that the research was conducted in the absence of any commercial or financial relationships that could be construed as a potential conflict of interest.

## Footnotes

1 http://blast.ncbi.nlm.nih.gov/Blast.cgi

2 https://img.jgi.doe.gov
