# Supplementary material for: Adaptation to Aerobic Environment of Lactobacillus johnsonii/gasseri Strains
Source: Front Microbiol. 2018 Feb 9;9:157. doi: 10.3389/fmicb.2018.00157 (PMC5811513; doi:10.3389/fmicb.2018.00157)
Supplement: Supplementary file 1 [file Table_1.docx]

**TABLE S1.** Results of *in silico* analysis of genes (query sequence from *Lactobacillus plantarum* WCFS1) involved in aerobic and respiratory metabolism and oxidative stress response in *Lactobacillus johnsonii* and *Lactobacillus gasseri* genomes from Integrated Microbial Genome and Microbiomes database (IMG/M).

| **Strains** | **Completeness level of genome** |  | ***pox3* (pyruvate oxidase)^a^** | | |  | ***ack2* (acetate kinase)** | | |  | ***loxL* (lactate oxidase)** | | |  | ***ndh* (NADH dehydrogenase)** | | |
| --- | --- | --- | --- | --- | --- | --- | --- | --- | --- | --- | --- | --- | --- | --- | --- | --- | --- |
|  |  |  | Occ  %^b^ | Locus Tag | %  ID |  | Occ  % | Locus Tag | %  ID |  | Occ  % | Locus Tag | %  ID |  | Occ  % | Locus Tag | %  ID |
| *L. johnsonii* |  |  | 100 |  |  |  | 100 |  |  |  | 42 |  |  |  | 14 |  |  |
| FI9785 | Finished |  |  | FI9785_1805 | 51 |  |  | FI9785_502 | 60 |  |  | FI9785_1781 | 50 |  |  | - | - |
| NCC 533 | Finished |  |  | LJ_1853 | 51 |  |  | LJ_0912 | 60 |  |  | - | - |  |  | - | - |
| DPC 6026 | Finished |  |  | LJP_1790c | 51 |  |  | LJP_1241c | 60 |  |  | - | - |  |  | - | - |
| N 6.2 | Finished |  |  | T285_09130 | 51 |  |  | T285_06205 | 60 |  |  | - | - |  |  | - | - |
| ATCC 33200 | Permanent Draft |  |  | HMPREF0528_0499 | 51 |  |  | Ga0106082_104191 | 62 |  |  | - | - |  |  | - | - |
| pf01 | Permanent Draft |  |  | PF01_01488 | 45 |  |  | PF01_00963 | 47 |  |  | - | - |  |  | PF01_00600 | 51 |
| 16 | Permanent Draft |  |  | Ga0081980_100422 | 52 |  |  | Ga0081980_1045116 | 47 |  |  | - | - |  |  | - | - |
| *L. gasseri* |  |  | 100 |  |  |  | 100 |  |  |  | 14 |  |  |  | 7 |  |  |
| ATCC 33323 | Finished |  |  | LGAS_1893 | 55 |  |  | LGAS_0431 | 51 |  |  | - | - |  |  | LGAS_1626 | 52 |
| 130918 | Finished |  |  | Ga0069304_121971 | 52 |  |  | Ga0069304_12681 | 61 |  |  | - | - |  |  | - | - |
| 224-1 | Permanent Draft |  |  | HMPREF9209_1596 | 45 |  |  | HMPREF9209_0556 | 61 |  |  | - | - |  |  | - | - |
| MV-22 | Permanent Draft |  |  | LBGGDRAFT_02078 | 52 |  |  | LBGGDRAFT_00613 | 46 |  |  | - | - |  |  | - | - |
| JCM 1131 | Permanent Draft |  |  | JCM1131DRAFT_00094 | 55 |  |  | JCM1131DRAFT_00560 | 51 |  |  | - | - |  |  | - | - |
| L32 | Permanent Draft |  |  | LGS32_00996 | 58 |  |  | LGS32_01619 | 60 |  |  | LGS32_02417 | 52 |  |  | - | - |
| SJ-9E-US | Permanent Draft |  |  | HMPREF0516DRAFT_01456 | 51 |  |  | HMPREF0516DRAFT_00713 | 51 |  |  | - | - |  |  | - | - |
| 202-4 | Permanent Draft |  |  | HMPREF0890_1112 | 45 |  |  | HMPREF0890_1123 | 61 |  |  | - | - |  |  | - | - |
| 2016 | Permanent Draft |  |  | M497_03520 | 45 |  |  | M497_08405 | 44 |  |  | - | - |  |  | - | - |
| SV-16A-US | Permanent Draft |  |  | HMPREF5175DRAFT_01637 | 51 |  |  | HMPREF5175DRAFT_00801 | 52 |  |  | - | - |  |  | - | - |
| K7 | Permanent Draft |  |  | LK7_00106 | 39 |  |  | LK7_00555 | 61 |  |  | - | - |  |  | - | - |
| JV-V03 | Permanent Draft |  |  | HMPREF0514_11618 | 52 |  |  | HMPREF0514_10278 | 61 |  |  | - | - |  |  | - | - |
| L3 | Permanent Draft |  |  | LGS03_01236 | 52 |  |  | LGS03_00282 | 61 |  |  | - | - |  |  | - | - |
| CECT 5714 | Permanent Draft |  |  | A131_64062 | 52 |  |  | A131_63426 | 44 |  |  | - | - |  |  | - | - |

| **Strains** | ***cydA* (cytochrome D ubiquinol oxidase subunit I)** | | |  | ***cydB* (cytochrome D ubiquinol oxidase subunit II)** | | |  | ***ubiE* (ubiquinone /manaquinone biosynthesis methyltrasferase)** | | |  | ***trxA* (thioredoxin peroxidase)** | | |
| --- | --- | --- | --- | --- | --- | --- | --- | --- | --- | --- | --- | --- | --- | --- | --- |
|  | Occ  % | Locus Tag | % ID |  | Occ  % | Locus Tag | %  ID |  | Occ  % | Locus Tag | %  ID |  | Occ  % | Locus Tag | %  ID |
| *L. johnsonii* | 100 |  |  |  | 100 |  |  |  | 100 |  |  |  | 100 |  |  |
| FI9785 |  | FI9785_1761 | 62 |  |  | FI9785_1762 | 59 |  |  | FI9785_116 | 53 |  |  | FI9785_496 | 61 |
| NCC 533 |  | LJ1810 | 62 |  |  | LJ1811 | 59 |  |  | LJ0053 | 53 |  |  | LJ0480 | 61 |
| DPC 6026 |  | LJP_1743 | 62 |  |  | LJP_1744 | 55 |  |  | LJP_0061 | 53 |  |  | LJP_0466 | 43 |
| N 6.2 |  | T285_08935 | 62 |  |  | T285_08940 | 59 |  |  | T285_00320 | 53 |  |  | T285_02375 | 55 |
| ATCC 33200 |  | HMPREF0528_0541 | 62 |  |  | HMPREF0528_0540 | 50 |  |  | HMPREF0528_0446 | 53 |  |  | HMPREF0528_0036 | 61 |
| pf01 |  | PF01_01448 | 64 |  |  | PF01_01449 | 55 |  |  | PF01_01575 | 54 |  |  | PF01_00129 | 61 |
| 16 |  | Ga0081980_10171 | 62 |  |  | Ga0081980_10172 | 62 |  |  | Ga0081980_10155 | 41 |  |  | Ga0081980_104717 | 38 |
| *L. gasseri* | 100 |  |  |  | 100 |  |  |  | 100 |  |  |  | 100 |  |  |
| ATCC 33323 |  | LGAS_1841 | 64 |  |  | LGAS_1842 | 59 |  |  | LGAS_0051 | 53 |  |  | LGAS_0427 | 62 |
| 130918 |  | Ga0069304_121909 | 62 |  |  | Ga0069304_121910 | 55 |  |  | Ga0069304_1257 | 53 |  |  | Ga0069304_121562 | 56 |
| 224-1 |  | HMPREF9209_1524 | 51 |  |  | HMPREF9209_1525 | 55 |  |  | HMPREF9209_1667 | 54 |  |  | HMPREF9209_2113 | 57 |
| MV-22 |  | LBGGDRAFT_02156 | 50 |  |  | LBGGDRAFT_02155 | 40 |  |  | LBGGDRAFT_00757 | 41 |  |  | LBGGDRAFT_01384 | 62 |
| JCM 1131 |  | JCM131DRAFT_00038 | 64 |  |  | J CM1DRAFT_00040 | 59 |  |  | JCM1131DRAFT_00780 | 43 |  |  | JCM1131DRAFT_00556 | 57 |
| L32 |  | LGS32_02557 | 50 |  |  | LGS32_02558 | 46 |  |  | LGS32_02172 | 54 |  |  | LGS32_01524 | 60 |
| SJ-9E-US |  | HMPREF0516DRAFT_01509 | 50 |  |  | HMPREF0516DRAFT_01508 | 40 |  |  | HMPREF0516DRAFT_00577 | 43 |  |  | HMPREF0516DRAFT_00804 | 45 |
| 202-4 |  | HMPREF0890_0547 | 58 |  |  | HMPREF0890_0546 | 40 |  |  | HMPREF0890_0917 | 54 |  |  | HMPREF0890_1432 | 62 |
| 2016 |  | M497_03250 | 65 |  |  | M497_03255 | 55 |  |  | M497_01865 | 53 |  |  | M497_04780 | 57 |
| SV-16A-US |  | HMPREF5175_01604 | 50 |  |  | HMPREF5175_01603 | 40 |  |  | HMPREF5175DRAFT_00605 | 53 |  |  | HMPREF5175DRAFT_01158 | 62 |
| K7 |  | LK7_00037 | 63 |  |  | LK7_00038 | 55 |  |  | LK7_01321 | 49 |  |  | LK7_01550 | 61 |
| JV-V03 |  | HMPREF0514_11680 | 53 |  |  | HMPREF0514_11679 | 40 |  |  | LGS32_01525 | 53 |  |  | HMPREF0514_10102 | 49 |
| L3 |  | LGS03_01171 | 62 |  |  | LGS03_01172 | 55 |  |  | LGS03_00422 | 45 |  |  | LGS03_00194 | 48 |
| CECT 5714 |  | A131_64178 | 50 |  |  | A131_54100 | 40 |  |  | A131_63987 | 53 |  |  | A131_35963 | 62 |

| **Strains** | ***trxB* (thioredoxin oxidase)** | | |  | ***gor* (glutathione reductase)** | | |  | ***gop* (glutathione peroxidase)** | | |  | ***npr* (NADH peroxidase)** | | |
| --- | --- | --- | --- | --- | --- | --- | --- | --- | --- | --- | --- | --- | --- | --- | --- |
|  | Occ  % | Locus Tag | %  ID |  | Occ  % | Locus Tag | %  ID |  | Occ  % | Locus Tag | %  ID |  | Occ  % | Locus Tag | %  ID |
| *L. johnsonii* | 100 |  |  |  | 100 |  |  |  | 0 |  |  |  | 0 |  |  |
| FI9785 |  | FI9785_517 | 51 |  |  | FI9785_107 | 47 |  |  | - | - |  |  | - | 61 |
| NCC 533 |  | LJ0501 | 51 |  |  | LJ0042 | 48 |  |  | - | - |  |  | - | 61 |
| DPC 6026 |  | LJP_0493c | 50 |  |  | LJP_0052 | 48 |  |  | - | - |  |  | - | 43 |
| N 6.2 |  | T285_02480 | 61 |  |  | T285_00250 | 47 |  |  | - | - |  |  | - | 55 |
| ATCC 33200 |  | Ga0106082_104129 | 51 |  |  | HMPREF0528_0013 | 44 |  |  | - | - |  |  | - | 61 |
| pf01 |  | PF01_01020 | 64 |  |  | PF01_01566 | 48 |  |  | - | - |  |  | - | 61 |
| 16 |  | Ga0081980_1047 | 65 |  |  | Ga0081980_10276 | 48 |  |  | - | - |  |  | - | 38 |
| *L. gasseri* | 100 |  |  |  | 85 |  |  |  | 7 |  |  |  | 28 |  |  |
| ATCC 33323 |  | LGAS_0447 | 43 |  |  | LGAS_0040 | 46 |  |  | - | - |  |  |  | 62 |
| 130918 |  | Ga0069304_121542 | 63 |  |  | Ga0069304_1244 | 47 |  |  | - | - |  |  |  | 56 |
| 224-1 |  | HMPREF9209_0485 | 62 |  |  | - | - |  |  | - | - |  |  |  | 57 |
| MV-22 |  | LBGGDRAFT_00549 | 62 |  |  | - | - |  |  | - | - |  |  | LBGG_00050 | 62 |
| JCM 1131 |  | JCM1131DRAFT_00577 | 43 |  |  | JCM1131DRAFT_00140 | 45 |  |  |  |  |  |  | - | 57 |
| L32 |  | LGS32_00137 | 68 |  |  | LGS32_02102 | 48 |  |  | LGS32_00080 | 59 |  |  | - | 60 |
| SJ-9E-US |  | HMPREF0516DRAFT_00773 | 54 |  |  | HMPREF0516DRAFT_01411 | 45 |  |  | - | - |  |  | HMPREF0516_00383 | 45 |
| 202-4 |  | HMPREF0890_1451 | 53 |  |  | HMPREF0890_0197 | 46 |  |  | - | - |  |  | - | 62 |
| 2016 |  | M497_08685 | 62 |  |  | M497_01805 | 46 |  |  | - | - |  |  | - | 57 |
| SV-16A-US |  | HMPREF5175DRAFT_00863 | 49 |  |  | HMPREF5175DRAFT_01591 | 45 |  |  | - | - |  |  | HMPREF5175_00403 | 62 |
| K7 |  | LK7_00572 | 43 |  |  | LK7_00152 | 46 |  |  | - | - |  |  | - | 61 |
| JV-V03 |  | HMPREF0514_10221 | 62 |  |  | HMPREF0514_11575 | 47 |  |  |  | - |  |  | - | 49 |
| L3 |  | LGS03_00225 | 62 |  |  | LGS03_01284 | 46 |  |  | - | - |  |  | - | 48 |
| CECT 5714 |  | A131_63398 | 43 |  |  | A131_64012 | 46 |  |  | - | - |  |  | A131_62641 | 62 |

^a^gene and gene product in parenthesis

^b^Occ: occurrence
